# Nursing students’ use of social media in their learning: a case study of a Canadian School of Nursing

**DOI:** 10.1186/s12912-022-00977-0

**Published:** 2022-07-22

**Authors:** Catherine M. Giroux, Katherine A. Moreau

**Affiliations:** 1 Faculty of Education, University of Ottawa, 145 Jean-Jacques Lussier Pvt, OttawaOttawa, ON K1N 9A7 Canada; 2 Faculty of Education, University of Ottawa, Ottawa, Canada

**Keywords:** Nursing students, Social media, Learning, Social constructivism, Third space

## Abstract

**Background:**

Social media has diverse applications for nursing education. Current literature focuses on how nursing faculty use social media in their courses and teaching; less is known about how and why nursing students use social media in support of their learning.

**Objectives:**

The purpose of this study was to explore how nursing students use social media in their learning formally and informally.

**Methods:**

This exploratory qualitative case study of a Canadian School of Nursing reports on the findings of interviews (*n* = 9) with nursing students to explore how they use social media in their learning. Data were analyzed using a combined deductive and inductive coding approach, using three cycles of coding to facilitate category identification.

**Results and conclusions:**

The findings demonstrate that participants use social media for formal and informal learning and specifically, as a third space to support their learning outside of formal institutional structures. Social media plays a role in the learning activities of nursing students studying both face-to-face and by distance. Accordingly, social media use has implications for learning theory and course design, particularly regarding creating space for student learning communities.

**Supplementary Information:**

The online version contains supplementary material available at 10.1186/s12912-022-00977-0.

## Background

Social media are online platforms that allow users to connect with other users, curate lists of connections, and interact with each other within the same online platform [1]. They have applications for both formal and informal learning in health professions education (HPE). Formal learning refers to planned educational experiences, such as courses or assignments [2] whereas informal learning refers to what is learned through extracurricular activities [3, 4]. With social media, formal learning may include such activities as using YouTube videos in class, while informal learning may involve students scrolling through Twitter and finding relevant learning content on their leisure time. Within the HPE literature, social media have been shown to facilitate electronic communication, networking, and real-time collaboration [5, 6]. They have also assumed key educational and communicative roles during the COVID-19 pandemic [7–9]. Furthermore, they continue to allow individuals to engage in independent, informal learning on their own terms and in places of formal education, work, or broader social circles [10]. Several studies demonstrated how social media can be used to facilitate clinical and professional performance tasks, question-and-answer sessions, and the exploration of complex topics collaboratively; social media can also provide professional learning opportunities and facilitate networking with international practitioners [11–14]. Moreover, instructors have used Twitter to provide students with formative feedback in assessment, stimulate reflection and sharing, share daily learning goals, hold journal clubs, notify students of recent topical publications, and orient learners to clinical sites and educational rotations [15–17]. The literature suggests that the connections that students make using social media can translate to opportunities for mentorship and scholarship [18]. Moreover, social media may also engage geographically dispersed individuals to create or share content, collaborate in groups, and ultimately form a virtual community [19, 20].

Within the nursing education literature, social media is well described as a tool selected by faculty for diverse formal teaching and learning purposes. For instance, several studies described using blogging to facilitate reflections as a teaching strategy for topics such as cultural competence, empathy, the therapeutic relationship, transitions to practice, and self-care [21–25]. The feedback system of the blogging interface provided students with opportunities to practice their reflection and problem-solving skills [26, 27]. Some studies used social media to simulate patient encounters or transition experiences for nurses [25, 28, 29]. For example, Thomas et al. used a blog to simulate a new nurse who had just transitioned to practice; the blog was written from the new nurse’s perspective to help final year nursing students consider issues of delegating and supervising, adapting to change, risk and quality management, and legal and ethical issues as they prepared to transition to practice [25]. Students had to read the blog and post responses. Other studies focused on using Facebook or YouTube as collaborative and interactive tools to help nursing students prepare for examinations like the National Council Licensure Examination (NCLEX) [30–32]. Still, issues of professionalism arose in the nursing education literature, with some studies noting concerns about students’ online behaviour and potential implications for their reputations and licensure [5, 33]. A 2021 narrative review found that learning about digital professionalism concepts as they relate to social media influenced how students behaved online [34]. Despite these potential professionalism implications, social media appears to be an effective tool to support formal learning in nursing education. A 2018 systematic review explored the effectiveness of using social media in nursing and midwifery education [35]. The authors found that the collaborative, interactive, and semi-synchronous nature of social media platforms may support knowledge and skill acquisition in nursing students.

Much of the extant undergraduate nursing education literature explores how social media is used in formal learning, specifically from the perspectives of the faculty who select the platforms to suit specific assignments or learning goals. Studies that focus on undergraduate students’ use of social media tended to explore specific platforms used and data analytics (i.e., hashtags used, number of views or shares). Less is known about how and why undergraduate nursing students themselves select social media platforms as an adjunct to their formal and informal learning activities. Thus, this exploratory qualitative case study aimed to address how and why undergraduate nursing students use social media to support their learning.

### Theoretical considerations

Social learning theories like social constructivism are appropriate for framing studies involving social media because they view learning as an active and collaborative process [36–38]. Social constructivism is based on three assumptions: (1) meanings are constructed by humans as they engage with the world they are interpreting; (2) humans engage with their world and make sense of it based on their historical and social perspectives; and (3) the basic generation of meaning is social, arising from the interaction with a human community [36]. Social constructivism claims knowledge is acquired when subjective meanings are created in interaction with others, drawing on material from previous experiences to guide learning [36–38]. This study was informed by social constructivism, which influenced our research questions, data collection instruments, and approaches to data analysis.

## Methods

### Research design

The objective of this study was to explore how students at one Canadian School of Nursing used social media to support their learning. We addressed this objective through an exploratory qualitative single case study. Yin [39] describes a case study as an empirical inquiry that investigates a contemporary phenomenon in depth within its real-world context even when the boundaries between the context and phenomenon may not be evident. Case studies comprise an all-encompassing method, which influences the logic of design, data collection techniques, and approaches to data analyses. Case study research is particularly useful for answering how and why questions; single case studies are appropriate for cases that are critical, unusual, revelatory, and longitudinal [39]. Our study site represented a critical case since the variety of program delivery methods and modalities were critically aligned with social constructivism. The study site also represented an unusual case, with four distinct program options – including a distance program – for students to achieve a Bachelor of Science in Nursing (BScN) degree. This was a unique program in Canada at the time of the study. The study site did not have any social media policies published to their public-facing website during the time of the study, nor did they have any public-facing references to using social media formally in their programs published on their website.

### Case study site and participants

This study took place at a small, relatively northern, Canadian university with a student population of approximately 5,090 students [40]. The School of Nursing, which includes 1191 students, offers four distinct, English-language, options for students to complete their BScN degree. These options include: 1) a standard four-year direct-entry nursing program; 2) an onsite Registered Practical Nurse (RPN) to BScN bridging program for students who previously obtained an RPN diploma and who are looking to subsequently obtain their BScN degree; 3) a part-time blended learning RPN to BScN bridging program for students currently working as RPNs who are looking to obtain their BScN; and 4) a second entry accelerated program for students who previously obtained an undergraduate degree. Only two of the nursing programs occur at the case study site itself. The second entry program is held in a large city to the south of the case study site. Additionally, students who partake in the RPN to BScN bridging program through blended learning live geographically dispersed throughout the province in which the case study site is located. Given the different program options, the participants in this study consisted of a mixture of face-to-face students and distance students. Additionally, due to the nature of the program options, some participants had pursued their nursing program as their first degree while others were already working as RPNs and had returned to school to obtain their BScN degree.

### Participant recruitment and data collection

Participants were purposively recruited from a previous study, which consisted of a digital artifact collection that explored what content nursing students shared to their Twitter, Facebook, and Instagram accounts related to learning [41]. The twenty-four nursing students who participated in our previous study were contacted by email and invited to participate in this qualitative case study exploring how and why nursing students use social media to support their learning. These students were identified as potential participants because they had confirmed using social media for learning and thus, would be information-rich interviewees for the present study. All potential participants were provided with a Participant Information Letter and Informed Consent form. The data for this study were collected using semi-structured interviews. All interviews were conducted virtually via Zoom in the Fall of 2019, using a semi-structured interview guide that had been developed based on the research questions, our theoretical framework, and the literature (refer to Additional file 1: Appendix). Prior to using the interview guide, it was piloted with two registered nurses. This pilot involved conducting two mock interviews and debriefing the interview guide with the participants to discuss the feasibility and appropriateness of the interview questions. The average interview length was 32 min, with the shortest being 21 min and the longest being 44 min long. We piloted the interview guide with two registered nurses prior to commencing the study. Each interview was audio-recorded and transcribed verbatim. Interview participation was incentivized with a $20 gift card to a local coffee chain.

### Data analysis

We took a combined deductive and inductive approach to coding and analyzing the interview transcripts. We sought to achieve theoretical sufficiency, which is the stage at which codes and categories manage new data without requiring further modification [42]. To do this, we conducted three cycles of coding in MAXQDA (v.18.2). In the first cycle, a preliminary codebook − which was informed by our research question, theoretical framework, and the literature − facilitated descriptive and process coding [43]. In the second cycle of coding, we each independently inductively coded the data using both process coding and in vivo coding (i.e., using the participants’ own words) and compared and discussed our coding. In the third cycle of coding, we grouped these summaries into categories, themes, or constructs [43]. A combination of matrices and networks visually displayed the data and facilitated category identification [43, 44].

### Reflexivity and trustworthiness

Neither author is a Registered Nurse nor is affiliated with the case study site. Both authors have expertise in conducting educational research within the health professions and were involved in the study conceptualization, data collection, and analysis. We also took steps to ensure that our analyses were credible, dependable, confirmable, and transferable [45, 46]. To establish credibility, we engaged in member-checking, wherein we provided the participants a copy of their interview transcripts so that they could ensure that their statements were accurately represented during transcription [45, 47]. We also engaged in peer debriefing. In terms of dependability, each of us inductively coded the data, compared our coding, and discussed and resolved any inconsistencies. In addition, we used audit trails as a strategy to ensure confirmability. These audit trails documented each of our decisions made during the research process and would allow an independent auditor to follow our steps and decisions to establish the same conclusions about the data. Lastly, through purposeful sampling and information-rich interviewees, we were able to obtain thick descriptions of how and why the students use social media to support their learning. We also included detailed descriptions of our research processes. This level of description allows others to judge the contextual similarity and transferability of our study findings.

### Ethical considerations

The interviews received formal institutional ethical approval (S-08–18-921) and approval from the study site (101916) in August 2019. We reviewed the informed consent form with each participant prior to commencing the interview and addressed any questions that they had. All participants verbally consented to participate in an interview and participants’ consent was recorded using Zoom video conference software, in accordance with our research ethics board approval.

## Results

Nine nursing students (*n* = 9) participated in the individual interviews. All participants were female and ranged in age from 18 to 49. Five participants attended classes online in a blended program format that occurred by distance and four participants attended classes face-to-face. The findings demonstrate that participants used social media in numerous ways for both formal and informal learning purposes. Table 1 provides a thematic overview of how the nursing student participants use social media in support of their learning.

**Table 1:** Thematic overview of how and why study participants used social media to support their learning

Preferred Social Media Platforms	Frequency
YouTube	9
Facebook	8
Instagram	5
Twitter	1
WhatsApp	1
Figure 1	1
Pinterest	1
**Formal Uses of Social Media**	
Course assignments	7
Clarifying course content	5
Course-related sharing	5
Studying for exams	3
Patient education	3
**Informal Uses of Social Media**	
Connect with peers and the nursing community	9
‘Behind the scenes’ teaching and learning	8
Review clinical skills	6
Follow areas of interest	5
Scaffolding knowledge and accidental learning	4
**Why Use Social Media**	
Encouraged by programs and professors	8
Convenience	5
Engagement	4
**Why Not Use Social Media**	
Credibility and relevance of sources	7
Distraction	4
Professionalism	4
Accessibility	2
**Target Audiences**	
Friends	7
Family	3
General public	3

### Formal learning

Participants reported using social media for a variety of purposes pertaining to formal learning. Table 2 provides exemplary participant quotes outlining their experiences using social media for formal learning purposes.

**Table 2:** Participants’ experiences using social media for formal learning purposes

Theme	Participant Quotes
Sharing or clarifying course content	I know I post some of my own, like, kind of study notes or, like, little cheats and tricks and kind of things for certain difficult concepts and I, you know, share it with my classmates so whether or not they use it, I don’t know but I definitely share my own knowledge just to try to help other people (P6) Being distance ed, watching YouTube videos kind of grounds the, um, experience, I suppose. It’s adding that little extra. A lot of the stuff I do is reading and listening to teachers talk on like a video livestream, um, and it just feels like there’s an extra dimension that’s missing from online learning so watching YouTube videos kinda just fills in that extra little space you miss from … face-to-face teaching (P7)
Supplementing university services	[S]ometimes we use a classroom platform and the profs will post their PowerPoints on there and sometimes you get to class in the morning and the platform’s crashed for whatever reason. So, people will just, like, upload the file to Facebook and that way we can grab it off of there (P6)
Assignments and exams	We were placed with the health unit, um, and they had developed a new… mandate saying that for students in, I believe it was JK or SK had to get mandated vision treatment in school, kind of like they get, um, the dental screenings done in school. So our job was to kind of raise awareness and develop a Facebook campaign for their Facebook page to talk about different reasons why they should get vision screening and the impacts that it has on the student… so we developed four Facebook posts that had to kind of revolve around that or about funding options related to, like, if your student was recommended for more screenings after their initial one in school, um, then here is a post about different funding options, whether it’s OHIP or ODSP or anything like that (P1) [F]or exams we’ll form a collective group and each of us will list the chapters that we’ve covered, each one of us will take an assigned chapter to do a review of and then we’ll just post all the files, so we have summaries of everything. Um, yeah, it’s actually quite cool ‘cause then that way, you know, we did it for a midterm this term and it’s nice not having one person, not having to go through and do a full review of, you know, 21 chapters (P2)

#### Sharing and clarifying course content

Several participants reported using social media to share content related to their courses and to clarify course content. Participant 7 explained how “when it comes to having, like, a large quantity of information, I think Facebook’s a better platform for that. Um, you’re able to share different links, you’re able to share pictures, videos, news articles, almost anything, it seems now”. Two participants (Participants 05 and 07) shared contrasting experiences with using social media formally in their distance classes to clarify course concepts. In this instance, a professor had shared YouTube videos in the course. While Participant 7 appreciated the inclusion of videos, Participant 5 found this approach to be lazy, especially since the professor did not create the videos but rather included videos that, according to Participant 5, students would likely search for on their own to assist their learning.

#### Supplementing university services

Eight participants indicated that Facebook was a good platform to supplement or highlight existing university services. Participant 5 explained how, as a distance student, they used Facebook to learn about the services available to students, like the university’s tutoring service, which Participant 5 found helpful for statistics. Participant 6 described how they used Facebook specifically for sharing course resources, since that platform might be easier at times than the typical learning management system.

#### Assignments and exams

The participants described using social media as a mechanism to complete their course assignments and to study for course exams and the National Council Licensure Examination (NCLEX). Social media appeared to be involved in the process of completing assignments; it also appeared to be the product of some assignments. Participant 5 described how “any group projects that we have to do would, which in an online program seems a little silly to me to do group projects but, um, we’d have to find a way to collaborate and it was often over Facebook or that sort of thing”. Participant 1 described creating a social media campaign for their community health class to help parents access vision care for their school-age children. Participant 3 shared how they found posts about how to pass the NCLEX the first time shared to social media and Participant 2 explained how they use social media to review for their course exams by dividing course content up amongst a small group of students and sharing review notes and summaries online. Participant 2 also described using social media platforms like Reddit to understand the patient experience based on what patients choose to share to these sites.

### Informal learning

Participants indicated that they used social media for diverse informal learning purposes. Table 3 provides an overview of participants’ experiences using social media for informal learning.

**Table 3:** Participants’ experiences using social media for informal learning purposes

Theme	Participant Quotes
Creating community	I think [social media] helps, especially as a distance student because you don’t have that same in-person classroom connection to begin with that at least when there’s a platform, whether it’s my group on WhatsApp or the, you know, larger program, larger page group on Facebook that we can connect, we can ask questions, get responses within seconds, minutes, or hours. Um, there’s a feeling of camaraderie, um, that we wouldn’t have as distance students, um, just because we’re all having similar experiences, similar frustrations, similar challenges, um, and we’re sharing that so it’s very relatable (P2) I, we do have, um, more social [groups] for, um, like our year so like Nursing 2020 ‘cause I’ll be graduating this year. We have a Facebook platform where we’re able to, um, just talk about like questions in class or if we’re like, say we’re doing presentations we can post our notes for our presentation, um, so it’s like external resources that we’re going to use or if, even social things like buy your formal ticket or like, buy, we have nursing clothing orders. Or, hey, we’re doing this mixer, come and, like, join us for it. Things like that too so it’s kind of both…. like an academic purpose and also, like, social, get involved in the… nursing community (P1)
‘Behind the scenes’ knowledge sharing	We all share textbooks so I might buy a textbook for $20 and then at the end of the semester as long as the same edition is being used, I turn around and sell it for $20. Um, so we’re buying and selling and shipping textbooks all the time, um, which is pretty cool, rather than having to buy or rent them, so I mean there’s a cost benefit to it (P2) We were able to get a lot of insight from people who were ahead of us in school and then also provide guidance for people who were just starting so like people would ask which professors to take or which courses, um, were interesting, like for electives (P3) If there was somebody from the university who went and poked through, you know, went and poked through the Facebook page and posted the answers to certain questions like about study plans, about whatever for the students to see on the [school] platform, I think that would be beneficial for a lot of students (P5)
Scaffolding knowledge	Even having like, just platforms, like I said we have our Facebook group where we’re able to post both social and academic things which I think is so good because so many times people will be like, shoot, like this question I have about this project we’re doing, like does anyone else understand this? Or do we need to email the prof about it, kind of thing. Um, so I definitely think there are some huge benefits to it (P1)

#### Creating community

By far, students shared the value of social media for connecting with peers and the nursing community most frequently. Four participants spoke about how social media promotes connection between distance students. Participant 3 shared how social media “gives you that camaraderie that you’re missing in a classroom environment”. Four out of the five students who identified as an online student cited Facebook groups as being an important mechanism for connecting with their classmates who were spread throughout the province. One participant explained how “there is a group online, uh, [School Name] distance ed students so I use that quite a bit, um, just to get information on classes, um, what to expect from different professors, etc.” Five participants shared how social media helped them combat isolation in their learning. Participant 2 emphasized the importance of social media for connecting distance students, which was important since they did not have the same opportunities to meet their classmates face-to-face. Participant 1 described how participating in Facebook groups helped enhance both the academic and social aspects of their face-to-face learning experience. Participant 4 explained how “we find it’s been really useful, or even like finding little things, like finding rides to clinical and stuff like that. Like obviously not all of us can afford vehicles and stuff like that so just by helping each other out”. In fact, every participant who identified as a face-to-face student (*n* = 4) spoke about the importance of Facebook groups to their learning experience since they contributed to building community and sharing resources.

Similarly, six participants shared how social media connected them with the broader nursing community, outside of their programs and university. Participant 6 described how social media could connect people across the country with experts in the field and the resources they have created. Participant 9 explained how social media could be used to “take my learning outside of the avenues that can be addressed and presented within a program or any program, really. So, it allows you to kind of step outside of that, see what’s happening with other people, how they’re learning…” Participant 1 described how social media allows them to connect with the nursing community on both social and academic levels through sharing memes and experiences on platforms like Instagram and Facebook. Participant 3 shared how social media “probably gives a good, like, um, a good alternative perspective on things, other than the teacher’s”.

#### ‘Behind the scenes’ knowledge sharing

While the participants often spoke about content that was publicly available to them on social media, they also shared how they used social media for informal learning purposes in private or ‘behind the scenes’ ways. Five nursing students reported using social media to buy, sell, and share PDF versions of textbooks. Participant 5 shared how “people share PDFs of textbooks and all that sort of stuff, so it’s definitely saved me several hundred dollars”. Two participants expressed how they prefer social media to textbooks. Participant 9 described how their professors are “not the biggest towards textbooks because they said that the second they are printed they are out of date because of how fast information is changing within healthcare”. In this sense, Participant 9 found social media to be a helpful way to stay up to date with information that textbooks did not provide.

Similarly, three participants described using social media to discuss which professors were the best for each class. Participant 2 explained how “we often talk about which professors are the best for specific courses and so those classes tend to fill up really fast”. Participant 5 described how they use social media to ask questions about the university, share their perceptions of certain professors, and discuss which classes should or should not be taken at the same time. While eight of the nine interview participants actively participated in social media groups, three participants shared that the absence of faculty members in the social media groups could be problematic. Participant 2 suggested using more of the collaborative tools available on the university’s learning management system to eliminate some of the need for the social media groups and better include the faculty members. Participant 5 also found the absence of faculty members in the social media groups to be a problem and recommended involving faculty members in the private groups to correct misinformation and answer questions.

#### Scaffolding knowledge

In addition to sharing resources, three students indicated that the Facebook groups were essential for giving and receiving support throughout their nursing programs. Similarly, five nursing students shared how they use social media to review their clinical skills. Three participants used social media to review IV insertion. Participant 7 described how “I use Instagram, I follow someone, she, her, her tag is IV Queen or something like that, but she gives a lot of intravenous tips on how to insert IVs and how to care for them”. Participant 3 also described using YouTube videos to review IV compatibility. Participant 1 shared how they used YouTube to practice for their IV therapy lab. Participant 1 also described how “we have used some YouTube videos and tutorials and stuff in our labs where we’re able to view, like, for example just last week we were learning about central lines, um, so we looked at a video about how to do the dressing change for a central line”. Participant 1 also described how they use YouTube to learn about skills like ambulating patients prior to starting their surgical rotation so that they would understand what they were about to do on the rotation.

### Why use social media

The study participants presented several reasons to use social media in support of their formal and informal learning activities; similarly, they also presented several reasons to be cautious of using social media for these purposes. Table 4 presents an overview of exemplary participant quotations presented thematically.

**Table 4:** Participants’ reasons for using or not using social media in support of their formal and informal learning

Theme	Participant Quotes
Credibility and relevance of sources	I think sometimes, again, depending on the pages out there and what can be followed, I think my biggest thing would be the accuracy. So, for somebody that is very much using this for study purposes, um, for my schooling right now as well as the NCLEX coming up, a big thing for me was, um, how, how true is this information and how reliable is this? (P9) It really is a positive experience just because I’m able to critically think about something and be like, you know, is this even, you know, you see this random article on Facebook or YouTube or whatever and it’s like, does this really make sense? Is this really a legitimate source? Like, and it’s just second nature to be able to filter that kind of uncredible source out whereas again, people who haven’t grown up with that mindset would have a more difficult time probably (P6)
Professors and professionalism	I’m not very, I guess out front, at least for the Instagram page with kind of what’s going on in that sense, um, just because I do know there are some professionalism bodies that do kind of regulate and kind of watch what is being posted, specifically since I am still trying to get into the world of nursing, so I’m very conscious of that (P9) I try to keep social media and my profession kind of separate. Maybe once in a while I might post something, but it’s very rare. Like, I really ty to keep that stuff separate…. I find that nursing is a reputable profession. I really take pride in my job and, yeah. Not that I’m a party animal or anything, don’t get me wrong (P8) Like, we always get warned about, we can’t break confidentiality so we can never say something on any social media platform that discloses information about our patients or clinical experiences. Like, they said that we can talk about oh, I had a really good day at clinical but obviously that’s excluding everything about our patient and what we actually have done, and they warned us about getting expelled or anything, getting kicked out of the program. So, they obviously provide us with that precautionary teaching but other than that, there’s not really, we don’t have guidelines other than that (P6)
Convenience and accessibility	It’s an easy platform to share from. The interface usually works really well. It’s really not hard to go onto Facebook and actually instead of emailing someone, instead of logging online and opening my email and going from there, if I already have Facebook open, I just open up messenger and I can just share documents, just drag and click directly into that, it just is super easy, convenient thing to do (P7) You can’t disconnect from social media so that was one of the other things I’ve often experienced was that social media as a whole, I reach a point where it’s, because of news media or something that’s going on in the world, I just don’t want to deal with it but in order for me to get information for school and communicate with some of my classmates, I need that Facebook app, um, my account active in order to, you know, to access that (P2)
Engagement and distraction	I think any educational experience would be very beneficial, especially since kind of my generation and the rest going forward, we are very attached to social media so to be able to use that platform to help us learn and to communicate and kind of connect with us on that level since it is something that works well for us and that we are quite connected to (P9) You know when you kind of start drifting off in class and you just open up your browser and oh, there’s Facebook or oh, I’m just going to casually scroll through Instagram and it’s a distraction for sure. Or, even when you’re like, on campus between classes and you’re like, ‘oh, I’m going to finish this assignment’ and then you end up wasting an hour on Twitter or something (P6)

#### Credibility and relevance of sources

Seven participants discussed the credibility and relevance of the sources they found on social media. Participant 7 indicated that they find their friends and followers on social media do not tend to share a lot of content that “I don’t consider real, like the fake news, but it’s a lot of more credible sources, like major journal articles and stuff like that”. Participant 4 expressed that students are taking a risk in depending on social media rather than on their books and their notes. Other students, like Participant 6, emphasized the importance of developing critical thinking skills and being able to filter social media posts so that they could appropriately determine which sources were accurate or credible. Participant 8 indicated that relying on social media links provided by course professors was helpful since “you know if the instructors are posting those videos, then you know that they’re credible sources.”

#### Professors and professionalism

All nine nursing students shared how their professors, programs, and the importance of professionalism influenced their use of social media. Four participants shared that, perhaps with the exception of YouTube videos, their professors did not use social media in their teaching and discouraged its use by nursing students. Participant 6 explained that “social media is kind of shunned a lot in nursing because of that whole idea of don’t post anything, don’t share your clinical experiences and don’t, you know, breach privacy.” In some instances, participants reported that their professors did not use social media in their teaching but encouraged students to use it to complete course assignments, like learning portfolios. Participant 4 shared that “[the professors] really like the idea of us working together on things and utilizing each other to keep on track”, especially as it related to support during clinical placements.” Other participants described their professors incorporating podcasts, videos, and Reddit into their courses, which encouraged their use of social media for learning. Still, several participants expressed concerns related to professionalism on social media. Participant 3 explained how “I definitely avoid posting about like, things that involve substance use. I feel like there’s added pressure on people in certain, in various professions like healthcare and police that you should avoid because you’re supposed to uphold a certain image of the profession.”

#### Convenience and accessibility

Several participants discussed the convenience of social media. Two participants shared how it was easier for communication purposes than other methods (i.e., emails, calls, texts). Other participants described how social media provided a central repository for resources that could be easily accessed by classmates. However, Participants 3 and 5 highlighted some challenges to accessibility because of using social media for learning, notably poor internet connection and lack of transcriptions or alternative formats.

#### Engagement and distraction

Four participants shared how they found social media to be an engaging platform for learning in their nursing education. Participant 4 explained how social media helps highlight major class concepts in a variety of formats, which can be helpful for different learners. Several participants spoke about growing up with social media and how their previous experiences motivated them to use it as a tool to support their nursing education. Participant 6 explained how “I kind of grew up with technology and grew up with social media that I just know how to use it and know how to access it and don’t have a problem filtering out what I don’t need.” Despite how participants felt about social media’s potential for engagement, they also found it potentially distracting. This was a common theme amongst both face-to-face and distance students. Participant 2 described ending up in a “Facebook vortex, where I end up being on it for 2 h, not necessarily on that [program specific] group.”

## Discussion

The nursing student participants described multiple ways of using social media to support their learning. None of the students in this study described using social media for the same creative formal experiences as those published by Thomas et al. [25] wherein a course instructor developed a simulated student on Facebook for nursing students to interact with online. However, a couple of students outlined their experiences being required to use sites like Reddit to learn about the patient experience. Additionally, some participants described how they used social media to develop patient-oriented health advocacy campaigns for healthcare organizations, effectively demonstrating how social media is being used in their formal nursing education. The ways in which the nursing students use social media to support their formal learning demonstrate social media’s collaborative capacity for knowledge and information exchange for both on-campus and distance students [6, 48, 49]. The study participants used social media creatively to support their formal education; for instance, participants referenced program-specific Facebook groups where they could collectively decide on questions that they needed to ask their professors in class. This finding is consistent with that of Junco et al. [50], where they found social media to be a low-stress method for students to ask questions of their peers and educators.

Informally, participants indicated using social media to refresh their clinical skills before applying them in lab settings or during clinical rotations. While the findings of this study do not directly touch on the use of social media at the point-of-care, studies like that conducted by Hay et al. [51] demonstrate social media’s potential utility for enhanced clinical learning and patient safety. In this study, two participants described how they use social media, specifically YouTube videos, to help with patient education at the bedside. Moreover, the participants indicated that they took a cautious approach to using social media in their formal and informal learning out of concern for professionalism implications. Several students indicated that they had been warned about the repercussions of unprofessional online behaviour and had adjusted their behaviour accordingly. This finding is similar to that of a previous conducted narrative review by O’Connor et al. [34] that found that students were likely to change what content they shared using social media after learning about issues of professionalism.

Importantly, the participants in this study appeared to use social media as a third space. Aaen and Dalsgaard [52] describe the ‘third space’ as being one that emerges in boundaries or overlaps across spheres; they explain that third spaces emerge from a need for discourses that are unavailable or cannot be filled in existing settings. Participants described creating their own Facebook groups for their classes, cohorts, study groups, clinical groups, and programs. The students explained that faculty members were not present in their Facebook groups, although they did sometimes encourage students to join the groups to stay up to date on information. The participants shared that they used the groups to fill gaps in their education. Others described using the Facebook groups to create a sense of community they felt was missing in their distance learning. In fact, this study found that nursing students use social media in their education in several ways that are often hidden or ‘behind the scenes’. Aaen and Dalsgaard [52] found that Facebook formed a ‘third space’ that combined elements of academic, personal, and social communication that does not typically take place within conventional university structures or spaces. The findings of this study are similar in the sense that the nursing student participants used social media as a mechanism to collaborate, communicate, teach, and learn when traditional university avenues were unavailable to them.

This study has implications for learning theory in connected teaching and learning. Learning theories – and thus, approaches to teaching – have moved from behaviourist to constructivist in the age of technology [53]. Indeed, social learning theories like connectivism [54], Communities of Practice [55], and social constructivism [36] can reflect the realities of connected teaching and learning because they focus on collective learning and knowing in both physical and digital spaces. In the present study, social constructivism, specifically Vygotsky’s Zone of Proximal Development, is evident in the participants’ use of social media for formal and informal learning purposes. Vygotsky [56] defines the Zone of Proximal Development as “the distance between the actual development level as determined by independent problem solving and the level of potential development as determined through problem solving under adult guidance or in collaboration with capable peers” (p. 86). The participants in this study described using online social media groups to share information about course requirements, assignment information, and exam tips. Social media also appeared to be a method for students to consolidate, share, and engage in their learning as part of a larger social process. Several participants described experiences of scaffolding learning for their peers either within their own cohort or in cohorts behind them using social media groups. Scaffolding is a key component of Vygotsky’s Zone of Proximal Development and has applications for online course design; technical scaffolding allows learners to experience just-in-time instruction and be provided with resources to solve problems and generate new learning and understanding collaboratively online [57]. Thus, the online learning environment should provide learners with the resources, tools, and supports they need to build their own knowledge; scaffolding fades as learners develop their own knowledge and expertise [53].

### Implications for nursing education policy and practice

This study demonstrates that nursing students are using social media in their educational practices both formally and informally. This use of social media has implications for teaching and learning in nursing education. Faculty members must consider the purposes for which nursing students are using social media, especially informally. One finding of this study suggested that nursing students turned to social media to fill perceived gaps – both academic and social – in their learning experience. If faculty members and schools of nursing are aware that social media is being used by nursing students for formal and informal teaching and learning purposes, it can be leveraged to achieve specific competencies and learning objectives. Based on this study, we have highlighted recommendations for nursing education policy and practice.

At the policy level, professional and appropriate social media communication could be included as an educational competency in nursing education programs, if not already stated in guiding curriculum frameworks. The purpose of this recommendation is not to discourage social media use but rather to develop competent online communicators who are equipped to use social media for teaching, learning, advocacy, and knowledge translation purposes. At the institution level, increased training for both faculty members and students on digital literacies, identifying credible online sources, and managing misinformation could help ensure faculty and students feel equipped to use digital tools like social media effectively in their teaching and learning. Finally, at the course level, some participants valued using social media to extend their learning while others were more reluctant to use it; thus, approaching the use of social media with flexibility and allowing for choice is essential. Providing optional opportunities to extend learning may help encourage participation on social media and help students discover how social media platforms can be used as learning tools informally within the nursing profession.

### Limitations and future directions

This exploratory qualitative case study included individual semi-structured interviews with nursing students from one Canadian School of Nursing. Despite incentivizing interview participation, we were only able to recruit 9 of the 24 possible participants. It is also probable that those who participated were more interested in social media than those who did not participate. The interviews consisted of self-reported data from the perspectives of the participants. Although participants spoke about how their professors used social media in their courses, the professors’ perspectives were not included in this study, leaving a potential imbalance and area for future research. Moreover, our small qualitative sample did not allow for a stratified analysis based on the program delivery method. This type of analysis would be interesting to conduct with a larger, quantitative dataset. Lastly, while the interview guide included questions about the nursing student participants’ experiences using social media, it did not include questions about their cultural backgrounds. In future, it would be interesting to explore how students’ culture backgrounds influence how and why they use of social media.

## Conclusions

The nursing students in this study described and demonstrated using social media to support their formal and informal learning. The participants also used social media as a third space, one that is separate from the traditional confines of the university. Within this space, participants merged their personal and academic discussions to collaborate, share resources, mentor one another, and connect with nursing experts and professional institutions. This use of social media has implications for teaching and learning in nursing education, especially regarding learning theory, scaffolding, and online course design.

## Supplementary Information

**Additional file 1: Appendix.** Semi-Structured Interview Guide.

## Data Availability

Due to the qualitative case study nature of this research, the data generated and analyzed during the current study are not publicly available to maintain the anonymity of the study participants. Data are available from the corresponding author on reasonable request.
