# Supplementary material for: Nursing students’ use of social media in their learning: a case study of a Canadian School of Nursing
Source: BMC Nurs. 2022 Jul 22;21:195. doi: 10.1186/s12912-022-00977-0 (PMC9303836; doi:10.1186/s12912-022-00977-0)
Supplement: Supplementary file 1 — Additional file 1: Appendix. Semi-Structured Interview Guide. [file 12912_2022_977_MOESM1_ESM.docx]

**Appendix**

**Semi-Structured Interview Guide**

1. Tell me about your experiences using social media for learning purposes as a nursing student.
   1. Prompt: Are you involved in any closed social media groups that help with your nursing education? Tell me about your experiences with them.
   2. Prompt: Do you prefer using certain platforms for certain learning purposes? Can you tell me about it?
2. Tell me about the role that social media plays in learning in nursing education.
   1. Prompt: As a nursing student, have you ever used social media to teach somebody else something? Tell me about your experience.
   2. As a nursing student, have you ever used social media to teach yourself something? Tell me about your experience.
3. Tell me about your experiences using social media as a learning tool.
   1. Prompt: In your opinion, does your program incorporate social media into nursing education? How so?
   2. Describe how your professors use social media for teaching, if at all.
4. Tell me about how social media contributes to your learning both in class and out of class.
   1. Prompt: Describe how social media affects your learning environment.
   2. Prompt: Describe the impact of social media on your learning as a distance student.
   3. Prompt: Describe the impact of social media on your learning as an on-campus student.
5. What do you think are the benefits of using social media for learning as a nursing student?
6. What do you think are the challenges of using social media for learning as a nursing student?
7. What types of health- or nursing-related content do you like to share to your social media accounts for your friends/followers? How come?
   1. Prompt: As a nursing student, do you use social media for any public health-related education or advocacy activities? Tell me about your experiences.
   2. Prompt: Why do you choose to use social media for these purposes?
   3. Prompt: Who do you hope to target as the audience when you share health- or nursing-related content to your social media accounts?
8. How do the posts you see on social media influence your perception of the role of a nurse?
9. Is there anything else that you would like to share related to your use of social media for learning purposes in nursing education that I have not already asked you?
